# Experiences During a Psychoeducational Intervention Program Run in a Pediatric Ward: A Qualitative Study

**DOI:** 10.3389/fped.2018.00124

**Published:** 2018-05-01

**Authors:** Paula Magalhães, Rosa Mourão, Raquel Pereira, Raquel Azevedo, Almerinda Pereira, Madalena Lopes, Pedro Rosário

**Affiliations:** ^1^School of Psychology, University of Minho, Braga, Portugal; ^2^Pediatric Service, Hospital of Braga, Braga, Portugal

**Keywords:** hospitalization, school-age children and adolescents, hospital psychoeducational services, hospital psychoeducational program, hospital-school linkage, school re-entry

## Abstract

Hospitalization, despite its duration, is likely to result in emotional, social, and academic costs to school-age children and adolescents. Developing adequate psychoeducational activities and assuring inpatients' own class teachers' collaboration, allows for the enhancement of their personal and emotional competences and the maintenance of a connection with school and academic life. These educational programs have been mainly designed for patients with long stays and/or chronic conditions, in the format of Hospital Schools, and typically in pediatric Hospitals. However, the negative effects of hospitalization can be felt in internments of any duration, and children hospitalized in smaller regional hospitals should have access to actions to maintain the connection with their daily life. Thus, this investigation aims to present a psychoeducational intervention program theoretically grounded within the self-regulated learning (SRL) framework, implemented along 1 year in a pediatric ward of a regional hospital to all its school-aged inpatients, regardless of the duration of their stay. The program counts with two facets: the psychoeducational accompaniment and the linkage to school. All the 798 school-aged inpatients (*M*_*age*_ = 11.7; *SD*_*age*_ = 3.71; *M*_hospital stay_ = 4 days) participated in pedagogical, leisure nature, and SRL activities designed to train transversal skills (e.g., goal-setting). Moreover, inpatients completed assigned study tasks resulting from the linkage between the students' own class teachers and the hospital teacher. The experiences reported by parents/caregivers and class teachers of the inpatients enrolling in the intervention allowed the researchers to reflect on the potential advantages of implementing a psychoeducational intervention to hospitalized children and adolescents that is: individually tailored, focused on leisure playful theoretically grounded activities that allow learning to naturally occur, and designed to facilitate school re-entry after hospital discharge. Parents/caregivers highlighted that the program helped in the preparation for surgery and facilitated the hospitalization process, aided in the distraction from the health condition, promoted SRL competences, and facilitated the communication and linkage with school life. Class teachers emphasized the relevance of the program, particularly in the liaison between hospital and school, in the academic and psycho-emotional and leisure-educational support provided, and in smoothing the school re-entry.

## Introduction

Hospitalization, either lasting for long periods of time or just for a few days, is likely to be a difficult time for children and adolescents, having been identified by Clouser (1) as a time of potential crisis for children and their parents (2). While school-aged inpatients are away from their home, family members, and friends, they become deprived from their daily life normalcy [e.g., (3–5)], with literature reporting that hospital stays can be traumatic and stressful events in children's life [e.g., (6, 7)]. In fact, 60% of inpatient children experience negative impacts of hospitalization {e.g., nightmares, separation anxiety, or aggression towards authority [e.g., (3, 8, 9)]}, and many report feeling alone, sad, or bored, and sometimes even frightened by the hospital environment or procedures [e.g., (3, 10)]. Moreover, the age and the duration of the stay are two of the most impacting risk factors of the negative impacts of hospitalization [e.g., (9)]. Specifically, younger children are more vulnerable to the negative effects of hospitalization than older children or adolescents (4, 9). However, there is some controversy about the effects of the duration of the stay. Wright et al. (9), for example, described that after leaving the hospital, children with stays of two or 3 days exhibited more negative behaviors (e.g., aggression toward authority, fear from hospital and doctors) than children staying in hospital for shorter or longer periods of time. Nevertheless, despite diminishing over time and almost disappearing after 2 weeks, these impacts may last for longer periods (8, 11).

During hospitalization, children's life routines are interrupted and the return to normalcy after discharge may be challenging (5, 12) and may result in negative responses (13). Besides experiencing the negative side effects of hospitalization, school-age youngsters also miss classes, and lose educational and social opportunities to progress. Because of this, hospitalized school-age children and adolescents are more likely to show higher underachievement rates (14), higher risk of psychosocial problems (15), and a lower probability of concluding compulsory education (16) or entering higher education (17) than their non-hospitalized counterparts.

Acknowledging the need to appease the adverse effects of hospitalization, a growing number of pediatric healthcare professionals are becoming aware of the need to provide comprehensive psychosocial multidisciplinary care (e.g., psychologists, teachers) along with medical care (e.g., doctors, nurses) (5, 18). Among these professionals, teachers may play a crucial role because they are expected to treat hospitalized children as students and recognize that every setting is a potential learning place, including hospital wards. In fact, access to a good quality education constitutes a right that every child has, as explicit in the United Nations Convention on the Rights of the Child (19). Inclusively, a proposal for a Charter for Children in Hospital has already been devised [e.g., (20)]. Thus, hospitalized school-age youngsters deserve access to education while they are away from school (21). Not only education constitutes a right, but also this educational experience is likely to provide inpatients with some sense of normalcy, and help in their recovery (22). When hospitals provide inpatients with a connection to their school lives, it helps them maintain a sense of normalcy [e.g., (23)], it appeases the child and parents' worries related to school learning, and prepares the child for school re-entry after discharge. Moreover, education can provide youngsters with opportunities to maintain their connections with their lifeworld beyond the hospital wards, which can also ensure the support from their teachers, schools, and classmates (22). In fact, hospitalized children are more likely to be engaged in school when their counterparts and the class teacher preserve them in their minds in spite of their absence in school (24) (e.g., maintain contact sending drawings, recovering cards, text messages, or notebooks with school activities and homework to be completed). Consequently, the transition back to school is more successful when the contact with school and classmates is kept [e.g., (25)], including when the new information and communication technologies are employed [e.g., (26, 27)].

The provision of educational services for hospitalized children is, therefore, recommended worldwide [e.g., (28)]^1^^,^^2^ and acknowledged as vital. Many pediatric or large hospitals offer educational services and educational programs (21), commonly to inpatient children and adolescents with long stays or frequent admissions (e.g., children with chronic diseases, or severe health conditions). Moreover, the educational services provided are usually set up as Hospital Schools, in which the hospital teachers teach specific academic content, in a classroom (29). The United States (5), Canada (21), the United Kingdom (3), and Australia (30) are examples of countries with considerable experience running education programs in their pediatric wards. In spite of this long tradition, data on the type and quality of the educational services delivered is limited and difficult to access [e.g., 21]. Moreover, educational services tend to differ across hospitals [e.g., 5, 21], with great variability concerning organization, funding, and structure (e.g., staff, funds, and way of acting), so the comparison of the educational programs delivered is hindered.

In sum, the adverse effects of hospitalization on children [e.g., (5, 10)] and the negative outcomes of school absence due to poor health conditions [e.g., (21)] have long been receiving the attention of researchers. However, except for one study (31), to authors' knowledge, recent research related to the psychosocial impact of hospitalization on school-age children and adolescents is still focused on specific health conditions, such as chronic (e.g., asthma, diabetes) or extreme diseases (e.g., cancer) and traumatic injuries (e.g., brain injuries) [e.g., (2)], and evidence-based data on hospital-based school programs and clear guidelines, or examples of good practices, are still missing in the literature [e.g., (5, 32)].

### The current study

The main aims of the current study are twofold. First, to present a psychoeducational intervention program (PIP) designed to provide accompaniment to all hospitalized school-aged children and adolescents (hereafter children), regardless of the duration of their stay. Second, to describe the experiences and perspectives, as reported by parents/caregivers and school teachers, from the implementation of this PIP in the pediatric ward of a regional hospital over the course of 1 year.

The program aims to fill in several gaps in the literature related to the topic of educational interventions with hospitalized children. The first addresses the targets of this type of interventions. PIP differs from other programs as it goes beyond the scope of hospitalized children suffering from chronic or severe illnesses [e.g., (30)] and with lengthy hospital stays [e.g., (21)]. PIP provides educational opportunities even for the inpatients who stay in the hospital for just a few days by ensuring their educational accompaniment with activities tailored to the length of their stay.

Another gap in the literature that the program aims to fill in is how “education” is conceptualized in this setting. Traditionally, “education” in hospitals has been provided in the context of Hospital Schools, in which there is an attempt to somewhat follow the curriculum for each grade, through the teaching of classes, in a remedial approach, while attending to each child's needs (21, 29). In fact, this is recognized as an obstacle as teachers often see themselves under the obligation of teaching contents and school grades out of their area of expertize (5). Moreover, this approach is not fitted to short hospital stays because teachers are likely to fail to respond to children's instructional needs due to the short lapse of time. PIP differs from the traditional approaches as “education” is conceived not in terms of curriculum and subjects of each grade, but in terms of transversal, learning-to-learn skills that can be trained regardless of the school year each child is enrolled. This, by itself, turns the remedial approach into a preventive approach. Therefore, the goal of PIP is not to convey specific contents, but to equip children with skills that are of use in any grade and any school subject (e.g., strategic thinking, planning, monitoring, making summaries).

Furthermore, Hospital Schools tend to be incorporated into large central hospitals or large pediatric hospitals [e.g., (21)]. Such programs are expensive, particularly in terms of human resources; thus, centralizing this activity maximizes assets. Consequently, smaller regional hospitals, with few internments in the pediatric wards, miss the opportunity to have access to these programs that larger hospitals own, putting the children hospitalized in these services in a disadvantaged position (29). Therefore, PIP also aims to fill in this gap in the literature by attempting to build and describe a more economical program that could be easily implemented and replicated in small regional hospitals with small pediatric units. Complementarily, because this program is not rooted in a curriculum of a particular country, but on transversal learning-to-learn skills, it can easily be replicated in other countries.

Finally, contrary to other experiences, because the intervention developed is not merely an adaptation of the curriculum and teaching classes, it is not atheoretically grounded. That is, each activity developed has a clear rationale behind, based upon the framework of social cognitive theory of self-regulated learning (SRL).

#### Theoretical framework

The current intervention is grounded in the social cognitive framework that assumes that contextual variables and learning settings play an important role in students' motivation and self-regulation. The concept of SRL and its cyclical nature (33) are the theoretical background of the PLEE model (34, 35) used in the current intervention. SRL models address how individuals assume an agent role while learning. This agency is described by Bandura (36) as the ability of individuals to influence their own cognitive and behavioral functioning. The relevance of SRL is increasing in the literature as previous research shows that students who receive training in SRL strategies (e.g., goal setting, time management) engage more deeply in school tasks and show better academic outcomes (35, 37, 38). In fact, while self-regulating their learning, students make use of cognitive, and metacognitive processes to control cognition, motivation, learning environments, and behaviors (39), and this happens before, during, and after learning occurs (40). For instance, metacognitive training helps students in problem solving: identify and define the problem, select the appropriate strategy and monitor its efficacy, identify the obstacles, and ultimately find a solution (41).

The PLEE model consists of three phases: planning, execution, and evaluation (34, 35). This learning process is not only organized throughout each phase, but each phase is also embodied in the self-regulatory logic: in each phase, the cyclical logic is updated and contains elements of the three phases (42). The planning phase begins prior to performing the task. At this stage, it is expected that children are able to self-set goals and adopt learning strategies to help attain those same goals. The execution phase refers to the implementation of the plan designed and its monitoring. Finally, the evaluation phase consists in the analysis of the goal attainment. The achieved outcomes provide important information for the planning phase of the following tasks (43). In this sense, extant literature indicates that to improve learning, children need to be equipped with a repertoire of learning strategies to help them cope with the challenges of the learning process (37, 44) (e.g., promotion of self-questioning, problem solving).

In sum, this hospital intervention program anchored on SRL framework aims to train children on how to cope with the problems and obstacles resulting from their health condition without losing sight of their personal and academic goals.

## Methods

### Study participants

During the 1-year period of implementation of PIP, 1796 patients were hospitalized in the pediatric ward, of which 798 were school aged children staying an average of 4 days. Of these, 251 stayed for more than 3 days. Inpatients' age ranged between 6 and 17 (*M* = 11.7; *SD* = 3.71) and 315 were girls (39.5%). All school-aged children admitted in the ward during the period of implementation of PIP were offered the opportunity to participate in the activities. That is, during this 1-year-period, PIP was a regular service offered by the pediatric ward to all school-age children. All inpatients admitted during this period accepted to partake in the different activities developed and no drop-out occurred. However, inpatients were not participants of the current study. Participants of the present study included the parents/ caregivers of these patients and respective class teachers. The only inclusion criterion for parents/ caregivers to participate was that they were accompanying school-aged children, and for teachers was that they were involved in the linkage established between hospital and school (please see section Interventions and Procedure). Thus, during the PIP implementation, 185 (23%) parents/ caregivers of the 798 children were randomly asked to fill in a questionnaire; all accepted to participate in the survey (response rate of 100%). Regarding class teachers, 71 public and private schools were contacted by the hospital teacher, and an effective collaboration was established with 56 class teachers (response rate of 79%).

### Interventions and procedure

#### The pediatric ward

The inpatient unit is specialized and designed to monitor children's development and keep pace with the diagnoses and treatment of their health conditions (e.g., common illnesses, surgeries, orthopedic). Its organization and design is children focused: the setting has colorful walls that include pictures, characters, and sentences from children's story tales. The ward accommodates patients from new-borns (0) to 17 year olds, in a total of 30 beds. It includes two leisure rooms (one for younger and the other for older children) that provide opportunities for children to be distracted during the hospital stay. Autonomously, or with their parents or caregivers, children can spend time there drawing, playing table—or video-games, reading or simply talking. However, except for the PIP, the hospital does not organize educational activities tailored to the patients' clinical conditions, educational needs or to their families.

This pediatric ward is not suited to accommodate children with extreme health conditions. Thus, the average length of the stays is 4 days, despite varying according to patients' ages. For children aged zero to five, the stays range from 1 to 53 days and last 5 days on average (*SD* = 4.63). The stays of children and adolescents between the ages of 6 and 17 range from 1 to 58 days and last 4 days on average (*SD* = 4.17).

#### The research team and the program implementation

The PIP research team consisted of an educational psychologist, an intern in educational psychology, and a hospital teacher. All the research members have training on SRL (e.g., a master's degree or PhD on this topic), and the work developed was supervised by the program coordinator in weekly meetings. *First facet of the program: The psychoeducational accompaniment* was available and offered to each school-aged hospitalized child, without restrictions or inclusion criteria. It provided a set of psychoeducational activities tailored to the length of each individual hospital stay. On a daily basis, the educational psychologists working in the pediatric ward gathered the personal data (e.g., age, school name, school year) of the newly hospitalized children. Every child and their parents/caregivers were interviewed upon arrival, and the informed consents for participation in the intervention were collected. The purpose of the interview was to collect relevant information about the child, their family, and their school context to outline the individual intervention plan. The parents/caregivers were also informed about the possibility of establishing contact with the school (see below).

The educational psychologist contacted the nursing and medical staff in the ward whenever needed to gather information about the health and clinical condition of the children. They helped the research team understand each individual's clinical circumstances as well as the limitations and physical constraints of their illness. Moreover, when possible, the medical staff informed the program team about the anticipated length of stay of the inpatients.

Irrespective of the duration of the stay, the psychologists provided psychoeducational support to each child in the ward. The training on SRL and the recreational activities were provided individually or in small groups. Firstly, each child's educational needs were acknowledged from the information gathered in the parents'/caregivers' interview and performance indicators of developed tasks (e.g., digital memory games, paper and pencil activities). Secondly, a set of activities were designed to fit each child's specific educational needs (e.g., time management, attention focus, reading speed, emotional regulation) and general concerns (e.g., “I'm afraid of the surgery,” “Will I wake up in the middle of the surgery?”). Finally, the plan set for each child was implemented for the duration of the stay. Children practiced the following: the set of SRL strategies (e.g., outline a text or summarize), the PLEE model (e.g., discussion of educational scenarios where children have to apply the PLEE model to school activities, to healthy food or oral health habits, emotional regulation, strategic thinking and metacognition), and the transfer of the SRL strategies to daily life. These SRL competences were developed using recreational-pedagogical and teaching materials, serious games, and story-tools.

Regarding the use of serious games in the pediatric ward, these were played in tablets provided by a partner institution under the supervision of the educational psychologists. Serious games are games designed with purposes other than entertainment [e.g., (45)] and allow users to learn, teach, and train certain skills; favor attitude change, transmit knowledge, and can promote rehabilitation [e.g., (45–47)]. Chin and Tsuei (48) emphasized that digital games can be important tools for education, training, and health care. In PIP, serious games were used as a tool to help researchers learn about each child's difficulties and to assess specific competences, to train specific skills (e.g., attention, memory, and strategic planning) [e.g., (49, 50)], and to promote engagement with the task at hand [e.g., (51)].

Regarding the use of the story-tools, children staying for longer periods of time were the target of this implementation. “Yellow's Trials and Tribulations,” designed for children between 6 and 10 years of age [see 52)], and “Testas' Misadventures,” for children between 11 and 15 [see 52)], were the selected story-tools. Extant research [e.g., (52)] has shown that these story-tools provide school-aged children an opportunity to learn a broad repertoire of learning strategies and reflect on learning situations, ideas, and challenges, presented in a friendly context similar to that of their own. In this sense, the self-regulatory analysis of these narratives represented an opportunity for children to become aware of the SRL strategies used in the story plot and transfer that learning to their daily life (43).

*Second facet of the program: The linkage to school* aimed to smooth the transition back to school and help to overcome the missed school-time. This facet was activated whenever the foreseen stay was at least 3 days long, or, independent from the length of the stay, when the occasion of the school-year was critical (e.g., tests, examinations), or when the child or their parents displayed a great concern about the impact of the school absence on the learning progress and assessment. In the first personal contact, the child and parents were informed about this facet and asked for consent so that the liaison with the school was established. It was clarified that the aim was not to substitute the school work (e.g., classes of the subjects delivered by the hospital teacher) but rather help keep some linkage with the school's ongoing learning and facilitate catch up when returning to school. After the consent, the hospital teacher contacted the school to explain the aims of the PIP, and ask the student's class teacher for bilateral collaboration. The objectives of the connection and the aims of the program were explained during this contact. Details about the child's school achievement were also collected. Afterwards, all materials (e.g., notes, tests, worksheets) were sent to the hospital teacher (email contact was privileged), who printed and delivered them to the child. Whenever the clinical condition of the child allowed, the child participated in the tasks prescribed by the class teachers with the support of the hospital teacher. On a daily basis during the hospital stay, the hospital teacher reported to each child's respective school the tasks completed, the study support provided, and the developments of the health condition. Expected hospital discharge was also communicated at opportune moments; and soon after discharge the psychologist and the hospital teacher wrote a report on the steps taken with the school, the academic accompaniment provided, and the psychoeducational intervention developed with the child in the pediatric ward. The report was sent by email to the class teacher along with a satisfaction questionnaire to be completed (see Instruments and Measures section).

### Instruments and measures

#### Parents'/caregivers' questionnaire

This questionnaire, built for the purposes of the current research assesses the parents'/caregivers' perceptions about PIP and describes the linkage of the program with the school whenever it was observed by the parents/caretakers. It has two sections; the first consists of 11 items (e.g., “The program team motivated my child to learning;” “The contact of the hospital teacher with my child's school and class teacher was important and of relevance;” see Table 1) scored on a five point Likert scale ranging from 1 (completely disagree) to 5 (completely agree) (α = 0.88). The second section consists of an open question where parents/caregivers were invited to make a comment on their personal experience with PIP.

**Table 1:** Means and standard deviations for the responses of the Parents'/Caregivers' questionnaire.

**Item**	***M (SD)***
1. The support provided by the team psychologist(s) was relevant.	4.74 (0.58)
2. The psychoeducational support gave my child the opportunity to abstract from their health condition.	4.63 (0.55)
3. The psychoeducational support helped me personally (doubts, anxiety, uncertainty).	4.36 (0.64)
4. The psychoeducational support motivated my child to learning.	4.42 (0.64)
5. The use of *tablets* to train specific competences such as attention or memory is innovative.	4.54 (0.57)
6. The goals of the psychoeducational support provided, the procedures used and the confidentiality of the process were clearly explained.	4.59 (0.51)
7. The psychologists were careful with and sensible to my child's health condition.	4.73 (0.47)
8. I perceive the psychoeducational support provided has an important service during hospitalization.	4.76 (0.46)
9. I think that the collaboration with the school(s) is an important dimension of this program.	4.55 (0.61)
10. The role played by the hospital teacher in the contact with the class teacher of my child was important and of great relevance.	4.63 (0.49)
11. Having in mind your experience with this program how would you rate the psychoeducational support provided.	4.70 (0.49)

#### Teachers' satisfaction questionnaire

This questionnaire was designed for this investigation and has two sections. The first section addresses the level of satisfaction of the class teachers of the hospitalized children with the role played by the hospital teacher while the collaboration lasted. This section comprises four items, each assessing a different dimension (see Table 2). Participants answered on a five point Likert scale ranging from 1 (not at all satisfied) to 5 (completely satisfied) (α = 0.76). The second section consists on an open question where class teachers were invited to make a comment on their personal experience with PIP (“We would like you to comment on your own experience with the program”).

**Table 2:** Means and standard deviations for the responses of the Teachers' questionnaire.

**Item**	***M (SD)***
1. Approach in terms of adequacy, correction and professionalism.	4.80 (0.45)
2. Promptness of the information provided.	4.78 (0.47)
3. Relevance of the information provided.	4.76 (0.52)
4. Finalization of the process in terms of reporting the work developed with the child and communicating the date of hospital discharge.	4.74 (0.49)

### Ethics

This study was conducted in accordance with the recommendations of the ethics committee of the University of Minho and the ethics committee of the Hospital in which the intervention took place. Upon admission in the pediatric unit, all involved (i.e., children and parents/ caregivers) were debriefed regarding the PIP and those wishing to partake in the intervention gave written informed consent in accordance with the Declaration of Helsinki.

### Data analysis

Regarding the first section of the Parents'/Caregivers questionnaire, descriptive statistics was conducted using SPSS software. The analysis of the open question followed the standard approach for qualitative data. Participants' answers were transcribed verbatim to digital format for later coding. A thematic analysis was carried out following the steps indicated by Braun and Clarke (53). Answers were coded based on a codebook built following an inductive process at a semantic level (53). Data led to five emerging categories: (i) preparation for surgery and facilitation of the hospitalization process, (ii) distraction from the health condition, (iii) promotion of SRL competences, (iv) communication and linkage with school life, and (v) future expectations for PIP. To enhance the trustworthiness of the findings, two researchers conducted the thematic analysis by independently coding each answer. After each researcher coded the answers, the inter-rater reliability was assessed and they met to discuss discrepancies found in the coding process. Coding decisions were evaluated item per item and considered reliable if: (a) percent agreement ≥ 80% (54) and (b) κ ≥ 0.75 (excellent reliability) (55). Cohen's Kappa coefficient showed an inter-rater agreement of.93, which is considered almost perfect (55).

Regarding the first section of the Teachers questionnaire, descriptive statistics was conducted using SPSS software. The analysis of the open question followed the same approach as described for the parents'/caregivers' question, with the same two researchers coding each answer independently. Data led to five emerging categories: (i) relevance of the program, (ii) liaison between hospital and school, (iii) academic support provided, (iv) psycho-emotional and leisure-educational support provided, and (v) smoothing the school re-entry. The inter-rater reliability was assessed (Cohen's Kappa coefficient showed an inter-rater agreement of 0.95) and discrepancies found in the coding process were discussed and resolved.

## Results

### Parents'/caregivers' questionnaire

Most of the 185 parents/caregivers evaluated the intervention program delivered to their children very positively (see Table 1). Specifically, 135 (73%) parents/caregivers rated their experience and that of their hospitalized children with the maximum score (5 on a 1–5 Likert scale). Concerning the connection and communication between the hospital teacher and the school, 64% of the parents/caregivers whose children took part of this facet evaluated it with the maximum score, which indicates that PIP was understood as important and of relevance.

Regarding the open question, the five themes will be presented along with some illustrative quotes.

#### Preparation for surgery and facilitation of the hospitalization process. pip team helped in facilitating the children's understanding of the surgery process or hospital admission

“*It was really good when the psychologist explained to my child what was going to happen in the surgery, it pacified him.”* (Mother of an 11-year-old boy, who had a gall bladder surgery and stayed hospitalized for 2 days).

“*I think this program helps children to stay at ease with some difficulties and it helps them understand the reasons for why they have to stay hospitalized*.” (Mother of a 10-year-old girl, who had appendectomy and was hospitalized for 10 days).

#### Distraction from the health condition. this intervention was perceived as useful to distract children from the difficulties of their health conditions

“*Let this program continue because it helps children and their parents forget about the place where they are in as well as the pain that they are going through. Concerning my child, it was very good because she was isolated, and with the help of this program she could tune out and forget that she could not be together with the other children.”* (Mother of a 10-year-old girl, staying for 9 days due to appendectomy).

#### Promotion of self-regulated learning competences. the relevance of performing leisure activities designed to promote the SRL processes in the hospital setting was recognized

“*My child really liked the games he played in here with the psychologist, and he said that the activities helped him to get to know strategies for school and how to study for tests*.” (Mother of a 14-year-old boy, staying for 12 days due to urinary infection).

“*My son talked a lot about learning how to set short, medium, and long term goals with the psychologist*.” (Father of a 15-year-old boy, staying for 3 days due to vesicular stomatitis).

#### Communication and linkage with school life. the importance of the connection between hospital and school to minimize the impact of hospitalization on the inpatients academic learning was highlighted

“*The psychologists helped my son to tune out his struggles. With the hospital teacher, he could remember school things. For example, he did his tests for French and History which helped him to achieve higher grades at the end of the school term. The support was very important*.” (Mother of a 14-year-old boy, hospitalized for 59 days due to long term side effects of a traffic accident).

“*On the one hand, my daughter kept occupied at the hospital and, on the other hand, she had the opportunity of keeping connected to school. My daughter was still able to understand what was going on at school*.” (Mother of a 12-year-old girl staying in hospital for 5 days due to peritonsillar abscess).

#### Future expectations for PIP. the importance of pip for hospitalized children was highlighted and the program's continuation recommended

“*It is a program that must stay. It was very helpful to my child's recovery. Each and every day, my child was looking forward to more activities. This made the long-term hospital stay easier, and my child sustained motivation*.” (Mother of a 7-year-old boy, staying for 28 days due to pyomyositis).

### Teachers' questionnaire

Fifty-six questionnaires were sent and 46 (response rate of 82%) were returned completed. Data show that the connection between Hospital and School was positively assessed. Class teachers expressed their satisfaction with the way the hospital teacher conducted the communication process between the hospital and school during the period of hospitalization up to the student's hospital discharge (see Table 2). Thirty-three of these teachers (72%) gave the highest score in all the four items of the scale concerning the role played by the hospital teacher in the connection with the class teacher.

Regarding the open question, the five themes will be presented along with some illustrative quotes.

#### Relevance of PIP. it was stressed the importance of the work developed and how it added up

“*I consider this program totally relevant and with pertinent impact on the maintenance of the liaison between the school and the student by ensuring this linkage*.” (class teacher of a 12th grade student).

#### Liaison between hospital and school. it was underlined the role of the program in bridging the hospital with the schools, pointing out benefits of the connection, and identifying some of the actors involved in this bridging process

“*This program is really important to maintain the connection between the student and the school…I always kept in touch with the student while trying to collaborate with the other class teachers and the hospital team teacher who did an excellent job*.” (class teacher of an 8th grade student).

“*The collaboration … helped the student to feel more pacified about school while he was recovering from his illness…and the schools feel this collaboration brings major benefits to everyone*.” (class teacher of a 7th grade student).

#### Academic support provided. examples of academic tasks performed by the inpatients and opportunities children were given to keep up with their learning process were described

“…*while hospitalized, the student could keep on following the learning activities by means of this program because he had the supervision and support to complete the school tasks*.” (class teacher of a 9th grade student).

#### Psycho-emotional and leisure-educational support provided. the large scope of opportunities provided to inpatients, as well as its positive effects were reported

“…*the type of support provided to hospitalized children is extremely important to them and goes far beyond from occupying their free time… As a teacher and as a mother I am very grateful for such a program. This program minimizes the pernicious effects to a child of both school absence and staying in a hospital*.” (class teacher of an 8th grade student).

“… *(the program) enabled the student to keep on being cognitively stimulated*.” (class teacher of a 9th grade student).

“*The program proved to be very useful especially because X used to be a very anxious girl…(It) supported X and allowed her to cope with her school absence in a balanced way…*” (class teacher of a 11th grade student).

#### Smoothing the school re-entry. it was emphasized and pointed out the efforts and procedures accounting for a well-succeeded reintegration in school

“*In a simple and efficacious way, the student didn't stop keeping in contact with school life, where he returned after a week. A smooth transition was facilitated, and it allowed him to easily reintegrate into class work*.” (class teacher of a 4th grade student).

“…*(the program) speeds up the communication process between Student, School, and Hospital, and this way it enables an expedite process to the student's recovery from “lost” learning opportunities. It proves that a simple and brief contact can make a difference in cases where there is an urge to act as quickly as possible*.” (class teacher of a 10th grade student).

“*… the student reintegrated into the class work routine as she showed no difficulties in following her classmates and the teacher, and she had no need to interrupt or to make significant breaks during the explanation of the content*.” (class teacher of a 6th grade student).

Occasionally, all of the five themes were present in a single comment:

“*The work developed by this program is extremely relevant as it allows students to keep in touch with school while facilitating their return and their reintegration in the classes. The direct support of the hospital team teacher and the team psychologist, with the collaboration of the class teacher, prevented her from falling behind. She was able to study the content of her class work while she was hospitalized, and this facilitated her re-entry*.” (class teacher of a student attending the 3rd grade).

## Discussion

The current paper aimed to understand the experiences reported by parents/caregivers and class teachers of PIP in providing educational accompaniment to hospitalized school-age children. The program had two complementary facets (the psychoeducational accompaniment and the linkage to school) and both were put into practice in a pediatric ward over the course of 1 year. Overall, results showed that both parents/caregivers and teachers stressed the relevant role of PIP as an emotional, educational, and leisure-pedagogical support for the children during their hospital stay.

Extant literature shows that even for short periods of time, hospitalization interrupts school-age children's daily life routines and school attendance, with negative repercussions in children's developmental and educational processes (2, 5, 56). However, current models of educational intervention are usually implemented in large pediatric hospitals, in the context of Hospital Schools, where hospital teachers follow a remedial approach and teach specific subjects of the inpatients curriculum (29); or are only available to children with stays comprising long periods of time or with chronic diseases that imply repeated admissions (29). There is, therefore, a need to provide the large majority of inpatients who fail to meet these criteria with social support, allowing them to keep connected with their school and ongoing learning (57). PIP design acknowledges that the vast majority of the hospital stays are short and children are discharged to recover at home. Thus, PIP attempted a novel approach of a preventive nature. Instead of focusing on specific content or disciplines, the team focused on promoting transversal skills and SRL strategies required for an effective learning, regardless of the school grade (e.g., planning, strategic thinking, goal-setting). Additionally, children participated in normal daily life activities, acknowledged in the literature as one of the best ways to promote hospitalized children's mental health (58). Since literature shows that students with training in SRL are more likely to become involved in academic tasks and they can achieve higher results [e.g., (34, 37, 59)], PIP focused on the promotion of SRL competences and daily life activities. It was expected that this option could allow hospitalized school-age children to further understand themselves, reflect upon their school experiences, lower the negative emotional, social, and academic impact of hospitalization by the time of school re-entry, and contribute to an effective promotion of their psychosocial skills.

Although preliminary, the experiences gained reported by parents/ caregivers and class teachers suggest that it is possible to use the time of hospitalization to foster children's competencies, and further contribute to the recognition that the training of SRL strategies can occur in contexts other than school [e.g., (36, 60, 61)]. In fact, PIP's team learned while in the hospital that children engaged in educational tasks tend to be more focused on working competencies to grow, evolve and return to school, than on challenges and obstacles of the clinical condition. Finally, the close linkage with school through the collaborative work between the teachers from the school and from the hospital may have helped smooth the re-entry of participating children in school.

There were also indirect positive experiences that were reported by nurses and doctors informally to the team. Medical staff would occasionally mention the importance of the playful dimension of the tasks developed by PIP and of its positive impact on the recovery of the children. This experience is in line with extant research that shows that fear triggers inpatients' pain; so, activities that help appease pain are likely to help recovery (62). Additionally, medical staff would also comment that appeased parents/caregivers were less likely to interfere with their routines, and that, in fact, the inclusion of PIP in the pediatric ward routine allowed for a quieter clinical environment.

## Limitations, educational implications, and future research

Several limitations and challenges have been faced while running PIP. The sample used in this study is geographically limited, and this factor may have impacted on the experiences described. Future studies could consider applying PIP in other small and also in large hospitals in their pediatric units. Moreover, despite of all schools in the geographic area of the hospital having been previously informed of PIP's objectives, many of the class teachers contacted were not aware of this project. This may help explain some of the difficulties found in the first contact with class teachers when researches attempted to initiate the linkage between hospital and school.

Furthermore, the length of the hospital stay was an important constraint to the implementation of PIP. According to the literature, hospital stays are becoming shorter every day; children and adolescents are being sent home to recover before returning to school [e.g., (63, 64)]. Staying at home to recover after hospital discharge challenges educational programs such as PIP to translate the support given to school-age children from the hospital to their homes. Moreover, the short length of the stays in the pediatric ward imposes a continuous turnover of children admitted. Considering that educators have a short time window to design psychoeducational plans fitted to inpatients needs, this intense movement of children becomes challenging and demanding to the work of educators. In fact, this constraint has long been recognized in the literature (29). Inclusively, the PIP team had to adjust the activities in response to the ongoing particular needs of each patient (e.g., many children had to perform the activities while staying in bed due to their medical condition). These contingencies of the length of the hospitalization and the unexpected events (e.g., change of scheduled medical exams, sudden discharge) had an impact on the quality of the inpatients learning experiences. Therefore, future interventions could consider assessing PIP either at personal (e.g., anxiety related to the surgery interventions), social (e.g., anxiety related to school absence and the impact on school progress) or academic level (e.g., achievement gains of a close relationship between the hospital teacher and the class teacher). Moreover, researchers may wish to develop follow up strategies aiming to provide further information about the potential advantages of PIP when children have already returned to school.

Additionally, the particularities and characteristics of the hospital context and the low sensitivity to interventions other than that of clinical nature made the implementation of the intervention an important challenge. For example, despite the hospital board having agreed with the intervention, PIP struggled to find the resources to deliver the activities (e.g., school materials, equipped rooms for conducting the small-group activities). Moreover, despite the promising indicators of children's engagement in the tasks and parental encouragement for children to participate, the PIP team noticed that, in some cases, parents, teachers, and medical staff appeared to be somehow reluctant to cope with the enrolment of children in learning tasks. Prior research has stressed that a child's illness and hospitalization could mean a period of anguish and anxiety for many parents/caregivers. This is likely to hinder the children's school learning efforts because their parents are more focused on the treatment and recovery of their child rather than on the support for school work (2). Moreover, class teachers do not always understand the short absences of their hospitalized students as a menace to their learning. Class teachers may even devalue the impact of this brief school absence on students' learning: the teachers usually expect children to rapidly and naturally “catch up” when they return to school (21). School administrators could consider helping teachers reflect upon the emotional and academic negative impact of hospitalization, even if it lasts for a short period of time. During PIP implementation, some class teachers were more focused on the health condition and recovery of their students than on the potential advantage of the hospital-school interaction for the children development. To illustrate this aspect, after hospital discharge, when a long stay at home for recovery was expected, the PIP's hospital teacher encouraged schools to use other means to maintain the child's connection with school, school learning, and peers (e.g., attend classes using Skype). Despite the knowledge that the new technologies of information and communication may be a useful and efficacious tool to provide the interchange of information, namely about the child's school achievement and hospital condition (26, 27), the suggestion made by the team's hospital teacher to use these tools was not accepted by the class teachers. Hospital and school administrators could consider working together to set and share responsibility regarding the educational programs offered inside hospitals because school engagement is important to children's overall quality of life (40). The communication between these two worlds (22) needs to be effective to improve the quality and effectiveness of the educational services provided to school-age hospitalized children and adolescents (5).

## Conclusion

The results of the implementation of PIP, as experienced by parents/caregivers and class teachers, suggest that the psychoeducational support provided to hospitalized school-age children was meaningful in different domains. Parents/caregivers highlighted that PIP helped in the preparation for surgery and facilitated the hospitalization process, aided in the distraction from the health condition, promoted SRL competences in their children, and facilitated the communication and linkage with school life. Regarding class teachers, they emphasized the relevance of the program, particularly in the liaison between hospital and school, the academic and psycho-emotional and leisure-educational support provided to the hospitalized children, and in smoothing the school re-entry.

The authors' current experience, which corroborate Hopkins et al. (22) work, and the experiences reported by parents/caregivers and teachers, all stress the need to understand school-aged hospitalized children as learners. In fact, despite of their health condition and learning particularities, hospitalized children proved to be willing and available to engage in new learning experiences. Providing children with learning opportunities and recognizing them as learners in continuous progress, even when they are hospitalized, is likely to reduce potential future educational risks, especially when the intervention is: (a) individually tailored, (b) focused on leisure playful theoretically grounded activities that allow learning to naturally occur, and (c) designed to facilitate school re-entry after hospital discharge.

## Author contributions

PM, RM, RP, RA, and PR contributed to the conception, data collection and analysis, and writing of this manuscript; PM and PR were responsible for the editing and major revisions of this manuscript; AP and ML contributed to the conception of this manuscript and made an important intellectual contribution in research design and manuscript revision. All authors approved of the final submitted version of this manuscript.

### Conflict of interest statement

The authors declare that the research was conducted in the absence of any commercial or financial relationships that could be construed as a potential conflict of interest.

## Footnotes

^1^European Association for Children in Hospital [homepage on the internet] [cited 2017 August 10]. Available online at: https://www.crin.org/en/library/organisations/european-association-children-hospital

^2^Charter for Children in Hospital. (EACH 2002 / 2nd Edition 2006). *Ireland: Children in Hospital Ireland* (2006) 17p. Available online at: http://www.childreninhospital.ie/wp-content/uploads/2016/09/each-charter.pdf
